# μ-3-Thienylmalonato-κ^2^ *O* ^1^:*O* ^3^-bis­[triphenyl­tin(IV)]

**DOI:** 10.1107/S1600536808030043

**Published:** 2008-09-24

**Authors:** Minglei Yang, Handong Yin, Daqi Wang, Li Quan, Liansheng Cui

**Affiliations:** a College of Chemistry and Chemical Engineering, Liaocheng University, Shandong 252059, People’s Republic of China

## Abstract

The title compound, [Sn_2_(C_6_H_5_)_6_(C_7_H_4_O_4_S)], contains two molecules with similar conformations in the asymmetric unit. In each mol­ecule, the Sn atoms adopt a distorted tetra­hedral geometry arising from three C atoms of three phenyl rings and one O atom from the bridging 3-thienylmalonato ligand. The mol­ecules lie about inversion centers with the ligands facing each other, with C⋯O distances of 3.417 (10) and 3.475 (10) Å.

## Related literature

For biological activities of self-assembled organotin derivatives of carboxylic acid ligands, see: Gielen *et al.* (1988 ▶). For organotin carboxyl­ates, see: Win *et al.* (2007 ▶). For bond-length data, see: Allen *et al.* (1987 ▶).
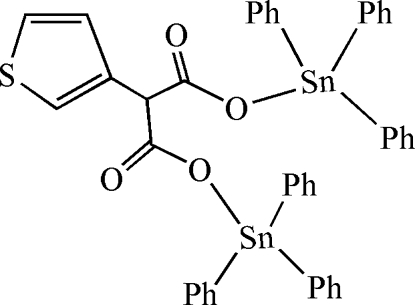


## Experimental

### 

#### Crystal data

[Sn_2_(C_6_H_5_)_6_(C_7_H_4_O_4_S)]
*M* *_r_* = 884.14
Monoclinic, 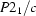

*a* = 11.7260 (13) Å
*b* = 21.905 (2) Å
*c* = 30.194 (3) Å
β = 93.542 (2)°
*V* = 7740.9 (15) Å^3^
*Z* = 8
Mo *K*α radiation
μ = 1.39 mm^−1^
*T* = 298 (2) K
0.28 × 0.12 × 0.09 mm

#### Data collection

Siemens SMART CCD area-detector diffractometer
Absorption correction: multi-scan (*SADABS*; Sheldrick, 1996 ▶) *T*_min_ = 0.698, *T*_max_ = 0.885
38418 measured reflections
13563 independent reflections
7274 reflections with *I* > 2σ(*I*)
*R*_int_ = 0.060

#### Refinement

*R*[*F* ^2^ > 2σ(*F* ^2^)] = 0.053
*wR*(*F* ^2^) = 0.131
*S* = 1.00
13563 reflections
901 parameters
H-atom parameters constrained
Δρ_max_ = 1.34 e Å^−3^
Δρ_min_ = −0.97 e Å^−3^

### 

Data collection: *SMART* (Siemens, 1996 ▶); cell refinement: *SAINT* (Siemens, 1996 ▶); data reduction: *SAINT*; program(s) used to solve structure: *SHELXS97* (Sheldrick, 2008 ▶); program(s) used to refine structure: *SHELXL97* (Sheldrick, 2008 ▶); molecular graphics: *SHELXTL* (Sheldrick, 2008 ▶); software used to prepare material for publication: *SHELXTL*.

## Supplementary Material

Crystal structure: contains datablocks I, global. DOI: 10.1107/S1600536808030043/pv2106sup1.cif

Structure factors: contains datablocks I. DOI: 10.1107/S1600536808030043/pv2106Isup2.hkl

Additional supplementary materials:  crystallographic information; 3D view; checkCIF report

## Figures and Tables

**Table 1:** Hydrogen-bond geometry (Å, °)

*D*—H⋯*A*	*D*—H	H⋯*A*	*D*⋯*A*	*D*—H⋯*A*
C6—H6⋯O6	0.98	2.48	3.417 (10)	160
C49—H49⋯O4	0.98	2.54	3.475 (10)	160

**Fig. 1.**
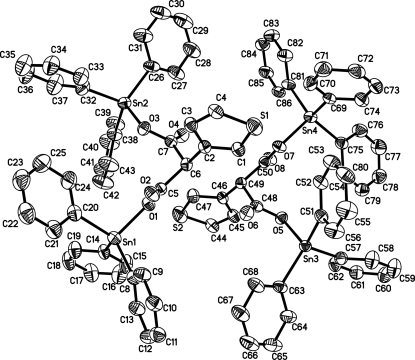
The molecular structure of the title compound, with the atom-numbering scheme; H atoms have been omitted for clarity. Displacement ellipsoids have been drawn at the 30% probability level.

**Fig. 2.**
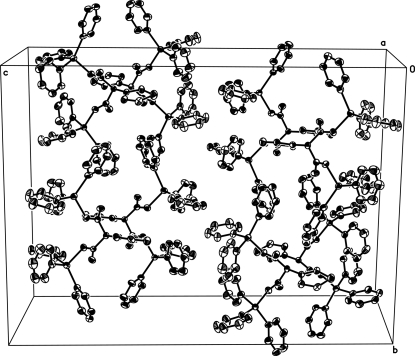
A packing diagram for (I). H atoms have been omitted for clarity.

## supplementary crystallographic information

### Comment

Self-assembled organotin derivatives of carboxylic acid ligands have been extensively studied due to their biological activities (Gielen *et al.*, 1988). Bi- or multidentate ligands containing O– or S-donors are often used to coordinate to tin centers. 3-Thiophenemalonic acid is a good bridging ligand that can be used to generate unexpected and interesting coordination polymers. We have therefore, synthesized the title compound, (I), and present its crystal structure in this article.

The asymmetric unit of the title compound, (I), contains two independent molecules (Fig. 1) with similar conformations wherein each 3-thiophenemalonicate is bonded to two triphenyl tin moities in a bidentate fashion. Each molecule consists of six phenyl and one (3-thiophenemalonicate) groups bonded to two tin atoms, where the bond lengths and angles are within normal ranges (Allen *et al.*, 1987). Each tin atom is bonded to three phenyl carbon atoms and an oxygen atom derived from the monodentate carboxyl group, thus, displaying a distorted tetrahedral geometry. The Sn—O distances in (I) lie in the range 2.042 (5) - 2.068 (5) Å and are close to the corresponding distances reported in organotin carboxylates (Yip *et al.*, 2007). Both the molecules in (I) lie about inversion centers with the ligands facing each other with C···O distances 3.417 (10) and 3.475 (10) Å (Table 1).

### Experimental

The reaction was carried out under nitrogen atmosphere. 3-Thiophenemalonic acid (1 mmol) and sodium ethoxide (2.2 mmol) were added to a solution of benzene (30 ml) in a Schlenk flask and stirred for 0.5 h. Triphenyltin chloride (2 mmol) was then added to the reaction mixture that was stirred for 12 h at 298 K. The resulting clear solution was evaporated under vacuum. The product was crystallized from a mixture of dichloromethane/methanol (1:1).

### Refinement

All H-atoms were positioned geometrically and refined using a riding model, with C—H = 0.93 and 0.98 Å for aryl and methine H-atoms, respectively, and *U*_iso_(H) = 1.2*U*_eq_(C). The final difference map showed the highest peak at 1.34 e Å^-3^ at a distance of 0.88 Å from Sn1 atom and was deemed meaningless.

### Crystal data

**Table:**

[Sn_2_(C_6_H_5_)_6_(C_7_H_4_O_4_S)]	*F*(000) = 3520
*M**_r_* = 884.14	*D*_x_ = 1.517 Mg m^−^^3^
Monoclinic, *P*2_1_/*c*	Mo *K*α radiation, λ = 0.71073 Å
Hall symbol: -P 2ybc	Cell parameters from 7272 reflections
*a* = 11.7260 (13) Å	θ = 2.2–25.3°
*b* = 21.905 (2) Å	µ = 1.39 mm^−^^1^
*c* = 30.194 (3) Å	*T* = 298 K
β = 93.542 (2)°	Block, colorless
*V* = 7740.9 (15) Å^3^	0.28 × 0.12 × 0.09 mm
*Z* = 8	

### Data collection

**Table:**

Siemens SMART CCD area-detector diffractometer	13563 independent reflections
Radiation source: fine-focus sealed tube	7274 reflections with *I* > 2σ(*I*)
graphite	*R*_int_ = 0.060
φ and ω scans	θ_max_ = 25.0°, θ_min_ = 1.4°
Absorption correction: multi-scan (SADABS; Sheldrick, 1996)	*h* = −13→13
*T*_min_ = 0.698, *T*_max_ = 0.886	*k* = −26→22
38418 measured reflections	*l* = −35→35

### Refinement

**Table:**

Refinement on *F*^2^	Primary atom site location: structure-invariant direct methods
Least-squares matrix: full	Secondary atom site location: difference Fourier map
*R*[*F*^2^ > 2σ(*F*^2^)] = 0.053	Hydrogen site location: inferred from neighbouring sites
*wR*(*F*^2^) = 0.131	H-atom parameters constrained
*S* = 1.00	*w* = 1/[σ^2^(*F*_o_^2^) + (0.0258*P*)^2^ + 39.3987*P*] where *P* = (*F*_o_^2^ + 2*F*_c_^2^)/3
13563 reflections	(Δ/σ)_max_ = 0.001
901 parameters	Δρ_max_ = 1.34 e Å^−^^3^
0 restraints	Δρ_min_ = −0.97 e Å^−^^3^

### Special details

**Table:**

Experimental. (yield 79%; m.p. 457–459 K). Analysis calculated (%) for C_43_H_34_O_4_SSn_2_ (Mr = 884.14): C, 58.36; H, 3.85. found: C, 58.39; H, 3.91.
Geometry. All e.s.d.'s (except the e.s.d. in the dihedral angle between two l.s. planes) are estimated using the full covariance matrix. The cell e.s.d.'s are taken into account individually in the estimation of e.s.d.'s in distances, angles and torsion angles; correlations between e.s.d.'s in cell parameters are only used when they are defined by crystal symmetry. An approximate (isotropic) treatment of cell e.s.d.'s is used for estimating e.s.d.'s involving l.s. planes.
Refinement. Refinement of *F*^2^ against ALL reflections. The weighted *R*-factor *wR* and goodness of fit *S* are based on *F*^2^, conventional *R*-factors *R* are based on *F*, with *F* set to zero for negative *F*^2^. The threshold expression of *F*^2^ > σ(*F*^2^) is used only for calculating *R*-factors(gt) *etc*. and is not relevant to the choice of reflections for refinement. *R*-factors based on *F*^2^ are statistically about twice as large as those based on *F*, and *R*- factors based on ALL data will be even larger.

### Fractional atomic coordinates and isotropic or equivalent isotropic displacement parameters (Å^2^)

**Table:**

	*x*	*y*	*z*	*U*_iso_*/*U*_eq_	
Sn1	0.39291 (5)	0.30434 (3)	0.61869 (2)	0.05001 (18)	
Sn2	0.41501 (5)	0.03143 (3)	0.64260 (2)	0.04940 (18)	
Sn3	0.10235 (5)	0.32048 (3)	0.853860 (19)	0.04442 (16)	
Sn4	0.10269 (5)	0.04933 (3)	0.88023 (2)	0.05154 (18)	
O1	0.3586 (5)	0.2391 (3)	0.66425 (18)	0.0508 (15)	
O2	0.5313 (5)	0.2599 (3)	0.69523 (19)	0.0593 (17)	
O3	0.4656 (5)	0.1078 (2)	0.67798 (18)	0.0515 (15)	
O4	0.3042 (5)	0.0936 (3)	0.7099 (2)	0.0593 (17)	
O5	0.0474 (5)	0.2441 (2)	0.81836 (18)	0.0503 (15)	
O6	0.2080 (5)	0.2566 (3)	0.7854 (2)	0.0599 (17)	
O7	0.1441 (5)	0.1130 (2)	0.83390 (19)	0.0537 (15)	
O8	−0.0279 (5)	0.0942 (3)	0.80156 (19)	0.0584 (16)	
S1	0.5913 (2)	0.17744 (13)	0.84609 (9)	0.0750 (8)	
S2	−0.0752 (3)	0.17549 (15)	0.64976 (9)	0.0894 (10)	
C1	0.4773 (8)	0.1876 (4)	0.8099 (3)	0.053 (2)	
H1	0.4056	0.1993	0.8184	0.063*	
C2	0.5009 (7)	0.1769 (4)	0.7671 (3)	0.045 (2)	
C3	0.6167 (7)	0.1589 (4)	0.7647 (3)	0.054 (2)	
H3	0.6492	0.1487	0.7384	0.065*	
C4	0.6769 (7)	0.1581 (4)	0.8065 (3)	0.054 (2)	
H4	0.7538	0.1481	0.8114	0.065*	
C5	0.4419 (8)	0.2319 (4)	0.6948 (3)	0.043 (2)	
C6	0.4124 (7)	0.1840 (4)	0.7290 (2)	0.042 (2)	
H6	0.3412	0.1970	0.7415	0.051*	
C7	0.3878 (8)	0.1243 (4)	0.7051 (3)	0.044 (2)	
C8	0.4121 (8)	0.3911 (4)	0.6478 (3)	0.053 (2)	
C9	0.5083 (8)	0.4063 (4)	0.6741 (3)	0.065 (3)	
H9	0.5636	0.3766	0.6807	0.078*	
C10	0.5245 (9)	0.4650 (4)	0.6912 (3)	0.065 (3)	
H10	0.5904	0.4745	0.7086	0.078*	
C11	0.4423 (9)	0.5087 (5)	0.6822 (3)	0.069 (3)	
H11	0.4515	0.5476	0.6942	0.082*	
C12	0.3465 (9)	0.4951 (5)	0.6555 (3)	0.071 (3)	
H12	0.2913	0.5248	0.6489	0.085*	
C13	0.3332 (8)	0.4365 (5)	0.6385 (3)	0.066 (3)	
H13	0.2687	0.4276	0.6201	0.080*	
C14	0.2416 (7)	0.2975 (4)	0.5780 (3)	0.054 (2)	
C15	0.1361 (9)	0.3088 (5)	0.5937 (3)	0.079 (3)	
H15	0.1321	0.3170	0.6238	0.095*	
C16	0.0358 (9)	0.3085 (6)	0.5673 (4)	0.095 (4)	
H16	−0.0337	0.3159	0.5795	0.114*	
C17	0.0401 (9)	0.2973 (5)	0.5232 (4)	0.084 (3)	
H17	−0.0265	0.2985	0.5048	0.101*	
C18	0.1410 (10)	0.2844 (5)	0.5060 (3)	0.082 (3)	
H18	0.1440	0.2761	0.4759	0.098*	
C19	0.2405 (9)	0.2838 (5)	0.5337 (3)	0.071 (3)	
H19	0.3090	0.2737	0.5216	0.086*	
C20	0.5382 (8)	0.2743 (5)	0.5870 (3)	0.062 (3)	
C21	0.6127 (9)	0.3166 (6)	0.5715 (3)	0.080 (3)	
H21	0.5962	0.3579	0.5741	0.096*	
C22	0.7099 (11)	0.2995 (7)	0.5524 (4)	0.095 (4)	
H22	0.7581	0.3287	0.5413	0.114*	
C23	0.7352 (11)	0.2401 (8)	0.5499 (4)	0.099 (4)	
H23	0.8022	0.2286	0.5371	0.119*	
C24	0.5664 (10)	0.2138 (6)	0.5845 (4)	0.086 (3)	
H24	0.5190	0.1843	0.5956	0.103*	
C25	0.6672 (11)	0.1966 (6)	0.5651 (4)	0.099 (4)	
H25	0.6865	0.1556	0.5627	0.119*	
C26	0.3880 (9)	−0.0466 (4)	0.6816 (3)	0.062 (3)	
C27	0.2886 (11)	−0.0522 (5)	0.7042 (3)	0.079 (3)	
H27	0.2365	−0.0201	0.7037	0.095*	
C28	0.2664 (11)	−0.1052 (6)	0.7274 (4)	0.090 (4)	
H28	0.2008	−0.1088	0.7430	0.108*	
C29	0.3427 (14)	−0.1514 (6)	0.7268 (4)	0.096 (4)	
H29	0.3270	−0.1872	0.7418	0.115*	
C30	0.4383 (13)	−0.1485 (5)	0.7060 (4)	0.094 (4)	
H30	0.4885	−0.1814	0.7071	0.113*	
C31	0.4644 (10)	−0.0952 (5)	0.6820 (4)	0.084 (4)	
H31	0.5311	−0.0928	0.6669	0.101*	
C32	0.5612 (8)	0.0244 (5)	0.6053 (3)	0.065 (3)	
C33	0.6702 (9)	0.0344 (5)	0.6222 (3)	0.076 (3)	
H33	0.6817	0.0450	0.6520	0.091*	
C34	0.7640 (10)	0.0294 (5)	0.5969 (4)	0.086 (3)	
H34	0.8365	0.0375	0.6099	0.103*	
C35	0.7529 (11)	0.0136 (6)	0.5551 (4)	0.100 (4)	
H35	0.8151	0.0140	0.5374	0.119*	
C36	0.6488 (12)	−0.0032 (7)	0.5385 (4)	0.111 (5)	
H36	0.6412	−0.0200	0.5102	0.133*	
C37	0.5521 (11)	0.0036 (6)	0.5624 (4)	0.104 (4)	
H37	0.4806	−0.0061	0.5492	0.124*	
C38	0.2677 (8)	0.0548 (4)	0.6027 (3)	0.061 (3)	
C39	0.1886 (10)	0.0093 (5)	0.5918 (4)	0.087 (4)	
H39	0.2013	−0.0301	0.6024	0.105*	
C40	0.0909 (10)	0.0220 (6)	0.5654 (4)	0.103 (4)	
H40	0.0401	−0.0092	0.5571	0.124*	
C41	0.0691 (10)	0.0800 (6)	0.5517 (4)	0.101 (4)	
H41	0.0021	0.0885	0.5348	0.121*	
C42	0.1414 (11)	0.1242 (6)	0.5621 (4)	0.106 (4)	
H42	0.1258	0.1637	0.5520	0.127*	
C43	0.2423 (10)	0.1123 (5)	0.5883 (4)	0.095 (4)	
H43	0.2923	0.1442	0.5958	0.114*	
C44	−0.1627 (8)	0.1929 (4)	0.6886 (3)	0.060 (3)	
H44	−0.2397	0.2024	0.6835	0.072*	
C45	−0.1034 (8)	0.1912 (4)	0.7308 (3)	0.058 (2)	
H45	−0.1369	0.2010	0.7570	0.070*	
C46	0.0129 (7)	0.1731 (4)	0.7290 (3)	0.046 (2)	
C47	0.0377 (8)	0.1639 (4)	0.6870 (3)	0.061 (3)	
H47	0.1098	0.1523	0.6789	0.073*	
C48	0.1238 (8)	0.2275 (4)	0.7913 (3)	0.046 (2)	
C49	0.0963 (7)	0.1663 (3)	0.7679 (2)	0.0404 (19)	
H49	0.1677	0.1517	0.7564	0.048*	
C50	0.0639 (8)	0.1212 (4)	0.8024 (3)	0.047 (2)	
C51	0.2515 (8)	0.2955 (4)	0.8928 (3)	0.052 (2)	
C52	0.2867 (8)	0.2356 (4)	0.8996 (3)	0.059 (3)	
H52	0.2433	0.2042	0.8863	0.071*	
C53	0.3825 (9)	0.2215 (5)	0.9249 (3)	0.072 (3)	
H53	0.4046	0.1809	0.9282	0.086*	
C54	0.4463 (9)	0.2658 (6)	0.9454 (4)	0.081 (3)	
H54	0.5111	0.2557	0.9632	0.097*	
C55	0.4152 (10)	0.3253 (6)	0.9398 (4)	0.092 (4)	
H55	0.4592	0.3559	0.9536	0.110*	
C56	0.3172 (10)	0.3405 (5)	0.9133 (4)	0.083 (3)	
H56	0.2964	0.3812	0.9095	0.099*	
C57	−0.0445 (8)	0.3288 (4)	0.8908 (3)	0.055 (2)	
C58	−0.0347 (9)	0.3431 (5)	0.9352 (3)	0.074 (3)	
H58	0.0371	0.3485	0.9495	0.089*	
C59	−0.1323 (10)	0.3495 (5)	0.9589 (4)	0.089 (4)	
H59	−0.1251	0.3590	0.9890	0.106*	
C60	−0.2371 (10)	0.3421 (5)	0.9387 (4)	0.075 (3)	
H60	−0.3016	0.3476	0.9546	0.090*	
C61	−0.2486 (8)	0.3268 (4)	0.8960 (3)	0.065 (3)	
H61	−0.3209	0.3208	0.8823	0.078*	
C62	−0.1539 (8)	0.3201 (4)	0.8726 (3)	0.061 (3)	
H62	−0.1635	0.3092	0.8428	0.073*	
C63	0.1263 (8)	0.3989 (4)	0.8153 (3)	0.053 (2)	
C64	0.0494 (9)	0.4468 (4)	0.8156 (3)	0.069 (3)	
H64	−0.0165	0.4432	0.8311	0.083*	
C65	0.0715 (11)	0.5012 (5)	0.7924 (4)	0.083 (4)	
H65	0.0197	0.5334	0.7920	0.100*	
C66	0.1685 (12)	0.5059 (5)	0.7709 (4)	0.085 (4)	
H66	0.1828	0.5419	0.7559	0.102*	
C67	0.2468 (10)	0.4594 (5)	0.7705 (3)	0.080 (3)	
H67	0.3136	0.4639	0.7557	0.096*	
C68	0.2243 (9)	0.4058 (4)	0.7924 (3)	0.066 (3)	
H68	0.2759	0.3737	0.7918	0.079*	
C69	0.2540 (8)	0.0566 (4)	0.9221 (3)	0.053 (2)	
C70	0.3611 (9)	0.0471 (4)	0.9062 (3)	0.068 (3)	
H70	0.3664	0.0379	0.8764	0.082*	
C71	0.4600 (10)	0.0507 (5)	0.9332 (4)	0.081 (3)	
H71	0.5307	0.0433	0.9219	0.097*	
C72	0.4522 (10)	0.0654 (5)	0.9767 (4)	0.075 (3)	
H72	0.5186	0.0690	0.9951	0.089*	
C73	0.3510 (10)	0.0746 (5)	0.9936 (3)	0.073 (3)	
H73	0.3476	0.0840	1.0235	0.087*	
C74	0.2491 (9)	0.0702 (4)	0.9664 (3)	0.068 (3)	
H74	0.1789	0.0765	0.9783	0.082*	
C75	−0.0419 (8)	0.0825 (4)	0.9111 (3)	0.056 (2)	
C76	−0.1048 (9)	0.0454 (5)	0.9364 (3)	0.077 (3)	
H76	−0.0823	0.0051	0.9409	0.092*	
C77	−0.2021 (10)	0.0667 (6)	0.9558 (4)	0.085 (3)	
H77	−0.2439	0.0409	0.9732	0.102*	
C78	−0.2353 (9)	0.1261 (5)	0.9489 (4)	0.077 (3)	
H78	−0.3008	0.1407	0.9612	0.092*	
C79	−0.1733 (9)	0.1631 (5)	0.9244 (3)	0.069 (3)	
H79	−0.1951	0.2037	0.9205	0.083*	
C80	−0.0795 (8)	0.1423 (4)	0.9055 (3)	0.060 (3)	
H80	−0.0389	0.1687	0.8882	0.072*	
C81	0.0827 (8)	−0.0379 (4)	0.8509 (3)	0.054 (2)	
C82	0.1691 (9)	−0.0810 (4)	0.8576 (3)	0.068 (3)	
H82	0.2363	−0.0717	0.8741	0.082*	
C83	0.1517 (10)	−0.1390 (5)	0.8386 (4)	0.075 (3)	
H83	0.2079	−0.1687	0.8429	0.090*	
C84	0.0546 (10)	−0.1522 (4)	0.8141 (3)	0.070 (3)	
H84	0.0451	−0.1908	0.8015	0.085*	
C85	−0.0295 (9)	−0.1097 (4)	0.8077 (3)	0.067 (3)	
H85	−0.0967	−0.1192	0.7912	0.081*	
C86	−0.0144 (9)	−0.0527 (4)	0.8258 (3)	0.061 (3)	
H86	−0.0713	−0.0235	0.8209	0.073*	

### Atomic displacement parameters (Å^2^)

**Table:**

	*U*^11^	*U*^22^	*U*^33^	*U*^12^	*U*^13^	*U*^23^
Sn1	0.0461 (4)	0.0518 (4)	0.0514 (4)	−0.0034 (3)	−0.0022 (3)	0.0055 (3)
Sn2	0.0507 (4)	0.0455 (4)	0.0509 (4)	0.0031 (3)	−0.0063 (3)	−0.0037 (3)
Sn3	0.0490 (4)	0.0388 (3)	0.0452 (3)	−0.0016 (3)	0.0008 (3)	0.0009 (3)
Sn4	0.0538 (4)	0.0426 (4)	0.0575 (4)	0.0015 (3)	−0.0018 (3)	0.0045 (3)
O1	0.047 (4)	0.059 (4)	0.045 (4)	−0.001 (3)	−0.003 (3)	0.007 (3)
O2	0.060 (4)	0.059 (4)	0.059 (4)	−0.015 (3)	−0.001 (3)	0.012 (3)
O3	0.049 (4)	0.053 (4)	0.053 (4)	−0.004 (3)	0.005 (3)	−0.009 (3)
O4	0.050 (4)	0.051 (4)	0.078 (4)	−0.013 (3)	0.012 (3)	−0.004 (3)
O5	0.048 (4)	0.047 (4)	0.056 (4)	−0.002 (3)	0.012 (3)	−0.003 (3)
O6	0.055 (4)	0.057 (4)	0.069 (4)	−0.016 (3)	0.011 (3)	−0.007 (3)
O7	0.053 (4)	0.051 (4)	0.056 (4)	0.001 (3)	−0.009 (3)	0.009 (3)
O8	0.057 (4)	0.055 (4)	0.062 (4)	−0.015 (3)	−0.005 (3)	0.010 (3)
S1	0.081 (2)	0.083 (2)	0.0584 (16)	−0.0057 (16)	−0.0113 (15)	0.0023 (15)
S2	0.101 (2)	0.103 (2)	0.0601 (18)	−0.0093 (19)	−0.0237 (17)	−0.0006 (17)
C1	0.051 (6)	0.059 (6)	0.048 (5)	−0.004 (5)	0.000 (4)	0.004 (5)
C2	0.046 (5)	0.043 (5)	0.044 (5)	−0.007 (4)	0.002 (4)	0.000 (4)
C3	0.046 (6)	0.066 (6)	0.050 (6)	−0.003 (5)	0.007 (5)	0.006 (5)
C4	0.041 (5)	0.061 (6)	0.061 (6)	−0.003 (4)	0.001 (5)	0.016 (5)
C5	0.047 (6)	0.040 (5)	0.040 (5)	−0.004 (4)	0.001 (4)	−0.002 (4)
C6	0.036 (5)	0.052 (5)	0.038 (5)	−0.003 (4)	−0.001 (4)	0.000 (4)
C7	0.043 (6)	0.045 (5)	0.043 (5)	−0.002 (4)	−0.003 (4)	0.001 (4)
C8	0.044 (6)	0.062 (6)	0.053 (6)	0.001 (5)	−0.002 (5)	0.005 (5)
C9	0.064 (7)	0.057 (7)	0.073 (7)	0.000 (5)	−0.010 (6)	0.005 (5)
C10	0.073 (7)	0.056 (7)	0.063 (6)	−0.011 (6)	−0.009 (5)	0.004 (5)
C11	0.080 (8)	0.058 (7)	0.068 (7)	−0.005 (6)	0.004 (6)	0.003 (5)
C12	0.072 (8)	0.060 (7)	0.080 (8)	0.013 (6)	−0.001 (6)	0.007 (6)
C13	0.063 (7)	0.068 (7)	0.066 (7)	0.002 (5)	−0.011 (5)	0.010 (6)
C14	0.039 (6)	0.078 (7)	0.044 (5)	0.000 (5)	0.003 (4)	0.008 (5)
C15	0.060 (7)	0.125 (10)	0.052 (6)	−0.002 (7)	−0.002 (6)	−0.014 (6)
C16	0.052 (7)	0.146 (12)	0.086 (9)	0.008 (7)	−0.003 (7)	−0.013 (8)
C17	0.052 (7)	0.130 (10)	0.068 (8)	−0.005 (7)	−0.012 (6)	0.007 (7)
C18	0.074 (8)	0.122 (10)	0.048 (6)	−0.010 (7)	−0.003 (6)	0.005 (6)
C19	0.055 (7)	0.100 (8)	0.059 (7)	−0.007 (6)	0.005 (5)	−0.001 (6)
C20	0.050 (6)	0.080 (8)	0.056 (6)	0.000 (6)	−0.001 (5)	0.006 (6)
C21	0.057 (7)	0.102 (9)	0.081 (8)	0.002 (7)	0.010 (6)	0.013 (7)
C22	0.069 (9)	0.128 (12)	0.088 (9)	−0.010 (8)	0.011 (7)	0.016 (9)
C23	0.068 (9)	0.133 (13)	0.098 (10)	0.007 (9)	0.019 (7)	0.003 (10)
C24	0.071 (8)	0.100 (10)	0.090 (9)	0.003 (7)	0.024 (7)	0.003 (7)
C25	0.083 (9)	0.106 (10)	0.112 (10)	0.018 (8)	0.021 (8)	0.004 (8)
C26	0.070 (7)	0.043 (6)	0.069 (7)	0.002 (5)	−0.026 (6)	−0.001 (5)
C27	0.099 (10)	0.061 (7)	0.074 (8)	−0.015 (6)	−0.028 (7)	0.011 (6)
C28	0.112 (11)	0.076 (9)	0.078 (8)	−0.019 (8)	−0.029 (7)	0.011 (7)
C29	0.120 (12)	0.068 (9)	0.094 (10)	−0.021 (9)	−0.044 (9)	0.014 (7)
C30	0.109 (11)	0.064 (8)	0.103 (11)	0.009 (8)	−0.046 (9)	0.006 (7)
C31	0.094 (9)	0.065 (8)	0.089 (8)	0.001 (7)	−0.041 (7)	−0.003 (7)
C32	0.060 (7)	0.082 (7)	0.051 (6)	0.009 (5)	−0.010 (5)	−0.020 (5)
C33	0.059 (7)	0.103 (9)	0.064 (7)	0.007 (6)	−0.005 (6)	−0.014 (6)
C34	0.058 (7)	0.117 (10)	0.082 (8)	0.002 (7)	0.000 (7)	−0.014 (8)
C35	0.076 (10)	0.135 (12)	0.088 (10)	0.003 (8)	0.009 (8)	−0.027 (9)
C36	0.091 (11)	0.171 (14)	0.071 (8)	0.012 (10)	0.008 (8)	−0.033 (9)
C37	0.072 (9)	0.157 (13)	0.081 (9)	0.011 (8)	−0.004 (7)	−0.029 (8)
C38	0.057 (6)	0.059 (6)	0.065 (6)	−0.002 (5)	−0.017 (5)	0.001 (5)
C39	0.079 (9)	0.077 (8)	0.101 (9)	−0.004 (6)	−0.028 (7)	0.012 (7)
C40	0.086 (10)	0.097 (10)	0.121 (11)	−0.014 (8)	−0.037 (8)	0.014 (9)
C41	0.077 (9)	0.100 (10)	0.121 (11)	0.000 (8)	−0.043 (8)	0.021 (9)
C42	0.096 (10)	0.085 (9)	0.130 (12)	−0.003 (8)	−0.039 (9)	0.029 (8)
C43	0.086 (9)	0.083 (9)	0.110 (10)	−0.010 (7)	−0.039 (8)	0.020 (7)
C44	0.040 (5)	0.074 (7)	0.066 (6)	−0.012 (5)	−0.002 (5)	0.016 (5)
C45	0.054 (6)	0.066 (6)	0.054 (6)	−0.011 (5)	0.000 (5)	0.011 (5)
C46	0.046 (5)	0.050 (5)	0.042 (5)	−0.006 (4)	0.002 (4)	0.003 (4)
C47	0.065 (7)	0.072 (7)	0.044 (6)	−0.004 (5)	−0.009 (5)	−0.006 (5)
C48	0.043 (6)	0.046 (5)	0.050 (6)	−0.001 (4)	−0.004 (4)	0.006 (4)
C49	0.041 (5)	0.041 (5)	0.040 (5)	−0.004 (4)	0.001 (4)	−0.003 (4)
C50	0.049 (6)	0.042 (5)	0.048 (6)	−0.002 (4)	0.005 (5)	−0.005 (4)
C51	0.052 (6)	0.057 (6)	0.047 (5)	−0.003 (5)	−0.004 (4)	0.004 (5)
C52	0.060 (7)	0.058 (6)	0.059 (6)	−0.002 (5)	−0.004 (5)	0.010 (5)
C53	0.066 (7)	0.073 (7)	0.075 (7)	0.008 (6)	−0.009 (6)	0.014 (6)
C54	0.067 (8)	0.091 (9)	0.082 (8)	0.005 (7)	−0.014 (6)	0.025 (7)
C55	0.081 (9)	0.083 (9)	0.107 (10)	−0.019 (7)	−0.030 (7)	0.002 (7)
C56	0.079 (8)	0.069 (7)	0.097 (9)	−0.009 (6)	−0.023 (7)	0.008 (7)
C57	0.057 (6)	0.055 (6)	0.053 (6)	0.000 (5)	−0.001 (5)	−0.002 (5)
C58	0.053 (7)	0.103 (9)	0.066 (7)	−0.011 (6)	−0.002 (6)	−0.015 (6)
C59	0.074 (9)	0.121 (10)	0.071 (8)	−0.009 (7)	0.012 (7)	−0.018 (7)
C60	0.060 (8)	0.090 (8)	0.079 (8)	0.002 (6)	0.018 (6)	−0.007 (7)
C61	0.053 (7)	0.073 (7)	0.068 (7)	0.004 (5)	−0.002 (6)	0.001 (6)
C62	0.057 (7)	0.075 (7)	0.050 (6)	0.002 (5)	−0.002 (5)	0.000 (5)
C63	0.063 (7)	0.041 (5)	0.054 (6)	−0.007 (5)	−0.014 (5)	0.005 (4)
C64	0.081 (8)	0.050 (6)	0.075 (7)	0.002 (5)	−0.011 (6)	0.006 (5)
C65	0.101 (10)	0.052 (7)	0.094 (9)	0.002 (6)	−0.023 (8)	0.009 (6)
C66	0.106 (11)	0.060 (8)	0.087 (9)	−0.022 (7)	−0.024 (8)	0.024 (6)
C67	0.085 (8)	0.072 (8)	0.080 (8)	−0.022 (7)	−0.009 (6)	0.019 (6)
C68	0.066 (7)	0.059 (6)	0.071 (7)	−0.015 (5)	−0.008 (6)	0.020 (5)
C69	0.056 (6)	0.050 (6)	0.051 (6)	0.012 (4)	−0.003 (5)	0.010 (5)
C70	0.070 (7)	0.077 (7)	0.057 (6)	0.014 (6)	−0.003 (6)	−0.008 (5)
C71	0.072 (8)	0.084 (8)	0.086 (9)	0.018 (6)	−0.009 (7)	−0.002 (7)
C72	0.074 (8)	0.075 (7)	0.071 (8)	0.011 (6)	−0.026 (7)	0.006 (6)
C73	0.086 (9)	0.076 (7)	0.054 (7)	0.007 (6)	−0.011 (6)	0.005 (5)
C74	0.071 (7)	0.073 (7)	0.061 (7)	0.009 (6)	0.001 (6)	0.010 (6)
C75	0.056 (6)	0.055 (6)	0.054 (6)	0.000 (5)	−0.007 (5)	0.003 (5)
C76	0.076 (8)	0.066 (7)	0.090 (8)	0.002 (6)	0.018 (7)	0.017 (6)
C77	0.074 (8)	0.087 (9)	0.096 (9)	−0.010 (7)	0.026 (7)	0.019 (7)
C78	0.065 (8)	0.086 (9)	0.081 (8)	0.008 (6)	0.010 (6)	−0.003 (7)
C79	0.066 (7)	0.068 (7)	0.072 (7)	0.011 (6)	0.000 (6)	0.002 (6)
C80	0.061 (7)	0.052 (6)	0.066 (6)	0.001 (5)	0.002 (5)	0.002 (5)
C81	0.059 (6)	0.037 (5)	0.067 (6)	0.000 (5)	0.002 (5)	0.011 (5)
C82	0.064 (7)	0.061 (7)	0.079 (7)	0.005 (5)	−0.006 (6)	0.005 (6)
C83	0.082 (8)	0.053 (7)	0.089 (8)	0.010 (6)	0.001 (7)	0.002 (6)
C84	0.087 (9)	0.046 (6)	0.078 (8)	−0.004 (6)	0.002 (7)	0.002 (5)
C85	0.077 (8)	0.052 (6)	0.070 (7)	−0.005 (6)	−0.015 (6)	0.005 (5)
C86	0.073 (7)	0.047 (6)	0.063 (6)	0.006 (5)	−0.002 (6)	0.012 (5)

### Geometric parameters (Å, °)

**Table:**

Sn1—O1	2.042 (5)	C36—C37	1.391 (15)
Sn1—C8	2.099 (9)	C36—H36	0.9300
Sn1—C14	2.100 (9)	C37—H37	0.9300
Sn1—C20	2.110 (10)	C38—C43	1.360 (13)
Sn2—O3	2.053 (5)	C38—C39	1.388 (13)
Sn2—C38	2.107 (9)	C39—C40	1.383 (14)
Sn2—C26	2.111 (10)	C39—H39	0.9300
Sn2—C32	2.114 (10)	C40—C41	1.354 (15)
Sn3—O5	2.068 (5)	C40—H40	0.9300
Sn3—C63	2.105 (9)	C41—C42	1.313 (15)
Sn3—C57	2.116 (9)	C41—H41	0.9300
Sn3—C51	2.119 (9)	C42—C43	1.407 (14)
Sn4—O7	2.055 (6)	C42—H42	0.9300
Sn4—C81	2.113 (9)	C43—H43	0.9300
Sn4—C75	2.113 (9)	C44—C45	1.412 (11)
Sn4—C69	2.120 (9)	C44—H44	0.9300
O1—C5	1.311 (9)	C45—C46	1.424 (11)
O2—C5	1.214 (9)	C45—H45	0.9300
O3—C7	1.313 (9)	C46—C47	1.334 (11)
O4—C7	1.205 (9)	C46—C49	1.488 (10)
O5—C48	1.302 (10)	C47—H47	0.9300
O6—C48	1.198 (9)	C48—C49	1.539 (11)
O7—C50	1.308 (9)	C49—C50	1.501 (11)
O8—C50	1.227 (9)	C49—H49	0.9800
S1—C4	1.662 (9)	C51—C56	1.374 (12)
S1—C1	1.688 (9)	C51—C52	1.386 (12)
S2—C44	1.651 (10)	C52—C53	1.355 (12)
S2—C47	1.703 (9)	C52—H52	0.9300
C1—C2	1.357 (11)	C53—C54	1.352 (13)
C1—H1	0.9300	C53—H53	0.9300
C2—C3	1.421 (11)	C54—C55	1.360 (14)
C2—C6	1.511 (10)	C54—H54	0.9300
C3—C4	1.409 (11)	C55—C56	1.399 (13)
C3—H3	0.9300	C55—H55	0.9300
C4—H4	0.9300	C56—H56	0.9300
C5—C6	1.525 (11)	C57—C58	1.376 (12)
C6—C7	1.512 (11)	C57—C62	1.377 (12)
C6—H6	0.9800	C58—C59	1.394 (13)
C8—C13	1.376 (12)	C58—H58	0.9300
C8—C9	1.381 (11)	C59—C60	1.347 (13)
C9—C10	1.395 (12)	C59—H59	0.9300
C9—H9	0.9300	C60—C61	1.328 (13)
C10—C11	1.374 (13)	C60—H60	0.9300
C10—H10	0.9300	C61—C62	1.362 (12)
C11—C12	1.375 (13)	C61—H61	0.9300
C11—H11	0.9300	C62—H62	0.9300
C12—C13	1.387 (12)	C63—C64	1.383 (12)
C12—H12	0.9300	C63—C68	1.385 (12)
C13—H13	0.9300	C64—C65	1.415 (13)
C14—C19	1.371 (12)	C64—H64	0.9300
C14—C15	1.374 (12)	C65—C66	1.348 (15)
C15—C16	1.380 (13)	C65—H65	0.9300
C15—H15	0.9300	C66—C67	1.372 (15)
C16—C17	1.359 (14)	C66—H66	0.9300
C16—H16	0.9300	C67—C68	1.381 (12)
C17—C18	1.351 (14)	C67—H67	0.9300
C17—H17	0.9300	C68—H68	0.9300
C18—C19	1.393 (13)	C69—C74	1.375 (12)
C18—H18	0.9300	C69—C70	1.387 (12)
C19—H19	0.9300	C70—C71	1.377 (13)
C20—C24	1.368 (13)	C70—H70	0.9300
C20—C21	1.375 (13)	C71—C72	1.361 (14)
C21—C22	1.362 (14)	C71—H71	0.9300
C21—H21	0.9300	C72—C73	1.335 (13)
C22—C23	1.338 (16)	C72—H72	0.9300
C22—H22	0.9300	C73—C74	1.411 (13)
C23—C25	1.342 (16)	C73—H73	0.9300
C23—H23	0.9300	C74—H74	0.9300
C24—C25	1.403 (15)	C75—C76	1.364 (12)
C24—H24	0.9300	C75—C80	1.387 (11)
C25—H25	0.9300	C76—C77	1.395 (14)
C26—C31	1.390 (13)	C76—H76	0.9300
C26—C27	1.392 (14)	C77—C78	1.371 (14)
C27—C28	1.389 (14)	C77—H77	0.9300
C27—H27	0.9300	C78—C79	1.343 (13)
C28—C29	1.352 (16)	C78—H78	0.9300
C28—H28	0.9300	C79—C80	1.351 (12)
C29—C30	1.318 (16)	C79—H79	0.9300
C29—H29	0.9300	C80—H80	0.9300
C30—C31	1.417 (15)	C81—C86	1.367 (12)
C30—H30	0.9300	C81—C82	1.390 (12)
C31—H31	0.9300	C82—C83	1.403 (12)
C32—C33	1.364 (12)	C82—H82	0.9300
C32—C37	1.369 (13)	C83—C84	1.350 (13)
C33—C34	1.382 (13)	C83—H83	0.9300
C33—H33	0.9300	C84—C85	1.363 (13)
C34—C35	1.308 (14)	C84—H84	0.9300
C34—H34	0.9300	C85—C86	1.368 (12)
C35—C36	1.341 (15)	C85—H85	0.9300
C35—H35	0.9300	C86—H86	0.9300
			
O1—Sn1—C8	111.8 (3)	C40—C39—C38	120.7 (11)
O1—Sn1—C14	98.6 (3)	C40—C39—H39	119.7
C8—Sn1—C14	112.0 (4)	C38—C39—H39	119.7
O1—Sn1—C20	106.6 (3)	C41—C40—C39	119.9 (12)
C8—Sn1—C20	113.8 (4)	C41—C40—H40	120.0
C14—Sn1—C20	112.9 (4)	C39—C40—H40	120.0
O3—Sn2—C38	107.4 (3)	C42—C41—C40	120.9 (12)
O3—Sn2—C26	114.7 (3)	C42—C41—H41	119.6
C38—Sn2—C26	111.7 (4)	C40—C41—H41	119.6
O3—Sn2—C32	96.8 (3)	C41—C42—C43	120.3 (12)
C38—Sn2—C32	112.1 (4)	C41—C42—H42	119.8
C26—Sn2—C32	113.1 (4)	C43—C42—H42	119.8
O5—Sn3—C63	114.9 (3)	C38—C43—C42	120.8 (11)
O5—Sn3—C57	96.0 (3)	C38—C43—H43	119.6
C63—Sn3—C57	111.2 (4)	C42—C43—H43	119.6
O5—Sn3—C51	107.4 (3)	C45—C44—S2	109.9 (7)
C63—Sn3—C51	112.6 (4)	C45—C44—H44	125.0
C57—Sn3—C51	113.6 (3)	S2—C44—H44	125.0
O7—Sn4—C81	110.7 (3)	C44—C45—C46	113.2 (8)
O7—Sn4—C75	107.2 (3)	C44—C45—H45	123.4
C81—Sn4—C75	115.1 (4)	C46—C45—H45	123.4
O7—Sn4—C69	97.5 (3)	C47—C46—C45	110.0 (8)
C81—Sn4—C69	112.6 (3)	C47—C46—C49	124.3 (8)
C75—Sn4—C69	112.1 (3)	C45—C46—C49	125.7 (8)
C5—O1—Sn1	112.9 (5)	C46—C47—S2	113.6 (8)
C7—O3—Sn2	111.0 (5)	C46—C47—H47	123.2
C48—O5—Sn3	110.3 (5)	S2—C47—H47	123.2
C50—O7—Sn4	113.5 (5)	O6—C48—O5	123.4 (8)
C4—S1—C1	93.3 (4)	O6—C48—C49	123.1 (8)
C44—S2—C47	93.3 (5)	O5—C48—C49	113.5 (7)
C2—C1—S1	113.1 (7)	C46—C49—C50	115.6 (7)
C2—C1—H1	123.4	C46—C49—C48	112.5 (7)
S1—C1—H1	123.4	C50—C49—C48	108.1 (6)
C1—C2—C3	110.4 (8)	C46—C49—H49	106.7
C1—C2—C6	122.5 (8)	C50—C49—H49	106.7
C3—C2—C6	127.1 (8)	C48—C49—H49	106.7
C4—C3—C2	112.6 (8)	O8—C50—O7	122.6 (8)
C4—C3—H3	123.7	O8—C50—C49	124.2 (8)
C2—C3—H3	123.7	O7—C50—C49	113.2 (7)
C3—C4—S1	110.5 (7)	C56—C51—C52	117.4 (9)
C3—C4—H4	124.8	C56—C51—Sn3	119.0 (7)
S1—C4—H4	124.8	C52—C51—Sn3	123.7 (7)
O2—C5—O1	123.5 (8)	C53—C52—C51	121.8 (9)
O2—C5—C6	124.9 (8)	C53—C52—H52	119.1
O1—C5—C6	111.6 (7)	C51—C52—H52	119.1
C2—C6—C7	112.2 (7)	C54—C53—C52	120.7 (10)
C2—C6—C5	114.4 (7)	C54—C53—H53	119.6
C7—C6—C5	108.4 (6)	C52—C53—H53	119.6
C2—C6—H6	107.2	C53—C54—C55	119.6 (10)
C7—C6—H6	107.2	C53—C54—H54	120.2
C5—C6—H6	107.2	C55—C54—H54	120.2
O4—C7—O3	121.6 (8)	C54—C55—C56	120.2 (11)
O4—C7—C6	123.8 (8)	C54—C55—H55	119.9
O3—C7—C6	114.6 (7)	C56—C55—H55	119.9
C13—C8—C9	117.3 (9)	C51—C56—C55	120.2 (10)
C13—C8—Sn1	121.0 (7)	C51—C56—H56	119.9
C9—C8—Sn1	121.5 (7)	C55—C56—H56	119.9
C8—C9—C10	121.6 (9)	C58—C57—C62	116.1 (9)
C8—C9—H9	119.2	C58—C57—Sn3	120.8 (7)
C10—C9—H9	119.2	C62—C57—Sn3	123.1 (7)
C11—C10—C9	119.4 (9)	C57—C58—C59	120.2 (10)
C11—C10—H10	120.3	C57—C58—H58	119.9
C9—C10—H10	120.3	C59—C58—H58	119.9
C10—C11—C12	120.2 (10)	C60—C59—C58	120.6 (11)
C10—C11—H11	119.9	C60—C59—H59	119.7
C12—C11—H11	119.9	C58—C59—H59	119.7
C11—C12—C13	119.1 (10)	C61—C60—C59	120.3 (11)
C11—C12—H12	120.4	C61—C60—H60	119.8
C13—C12—H12	120.4	C59—C60—H60	119.8
C8—C13—C12	122.3 (9)	C60—C61—C62	119.6 (10)
C8—C13—H13	118.8	C60—C61—H61	120.2
C12—C13—H13	118.8	C62—C61—H61	120.2
C19—C14—C15	115.0 (9)	C61—C62—C57	123.2 (9)
C19—C14—Sn1	122.9 (7)	C61—C62—H62	118.4
C15—C14—Sn1	122.1 (7)	C57—C62—H62	118.4
C14—C15—C16	123.5 (10)	C64—C63—C68	118.9 (9)
C14—C15—H15	118.2	C64—C63—Sn3	120.3 (8)
C16—C15—H15	118.2	C68—C63—Sn3	120.5 (7)
C17—C16—C15	119.1 (11)	C63—C64—C65	119.7 (11)
C17—C16—H16	120.4	C63—C64—H64	120.2
C15—C16—H16	120.4	C65—C64—H64	120.2
C18—C17—C16	120.0 (10)	C66—C65—C64	119.2 (11)
C18—C17—H17	120.0	C66—C65—H65	120.4
C16—C17—H17	120.0	C64—C65—H65	120.4
C17—C18—C19	119.5 (10)	C65—C66—C67	122.2 (11)
C17—C18—H18	120.2	C65—C66—H66	118.9
C19—C18—H18	120.2	C67—C66—H66	118.9
C14—C19—C18	122.7 (10)	C66—C67—C68	118.6 (11)
C14—C19—H19	118.6	C66—C67—H67	120.7
C18—C19—H19	118.6	C68—C67—H67	120.7
C24—C20—C21	118.2 (10)	C67—C68—C63	121.4 (10)
C24—C20—Sn1	122.1 (8)	C67—C68—H68	119.3
C21—C20—Sn1	119.4 (9)	C63—C68—H68	119.3
C22—C21—C20	121.6 (12)	C74—C69—C70	117.4 (9)
C22—C21—H21	119.2	C74—C69—Sn4	120.8 (7)
C20—C21—H21	119.2	C70—C69—Sn4	121.7 (7)
C23—C22—C21	119.1 (13)	C71—C70—C69	122.3 (10)
C23—C22—H22	120.4	C71—C70—H70	118.8
C21—C22—H22	120.4	C69—C70—H70	118.8
C22—C23—C25	122.2 (13)	C72—C71—C70	118.7 (11)
C22—C23—H23	118.9	C72—C71—H71	120.6
C25—C23—H23	118.9	C70—C71—H71	120.6
C20—C24—C25	119.8 (11)	C73—C72—C71	121.2 (11)
C20—C24—H24	120.1	C73—C72—H72	119.4
C25—C24—H24	120.1	C71—C72—H72	119.4
C23—C25—C24	119.0 (13)	C72—C73—C74	120.6 (10)
C23—C25—H25	120.5	C72—C73—H73	119.7
C24—C25—H25	120.5	C74—C73—H73	119.7
C31—C26—C27	119.2 (10)	C69—C74—C73	119.8 (10)
C31—C26—Sn2	120.4 (9)	C69—C74—H74	120.1
C27—C26—Sn2	120.2 (7)	C73—C74—H74	120.1
C28—C27—C26	120.6 (12)	C76—C75—C80	116.8 (9)
C28—C27—H27	119.7	C76—C75—Sn4	121.3 (7)
C26—C27—H27	119.7	C80—C75—Sn4	121.9 (7)
C29—C28—C27	118.3 (13)	C75—C76—C77	121.5 (10)
C29—C28—H28	120.9	C75—C76—H76	119.2
C27—C28—H28	120.9	C77—C76—H76	119.2
C30—C29—C28	123.7 (13)	C78—C77—C76	119.0 (10)
C30—C29—H29	118.2	C78—C77—H77	120.5
C28—C29—H29	118.2	C76—C77—H77	120.5
C29—C30—C31	119.9 (13)	C79—C78—C77	120.0 (11)
C29—C30—H30	120.0	C79—C78—H78	120.0
C31—C30—H30	120.0	C77—C78—H78	120.0
C26—C31—C30	118.4 (12)	C78—C79—C80	120.8 (10)
C26—C31—H31	120.8	C78—C79—H79	119.6
C30—C31—H31	120.8	C80—C79—H79	119.6
C33—C32—C37	115.1 (10)	C79—C80—C75	121.9 (10)
C33—C32—Sn2	124.0 (7)	C79—C80—H80	119.0
C37—C32—Sn2	120.6 (8)	C75—C80—H80	119.0
C32—C33—C34	122.7 (10)	C86—C81—C82	119.5 (9)
C32—C33—H33	118.7	C86—C81—Sn4	121.0 (7)
C34—C33—H33	118.7	C82—C81—Sn4	119.5 (7)
C35—C34—C33	121.2 (11)	C81—C82—C83	118.0 (9)
C35—C34—H34	119.4	C81—C82—H82	121.0
C33—C34—H34	119.4	C83—C82—H82	121.0
C34—C35—C36	117.9 (13)	C84—C83—C82	121.0 (10)
C34—C35—H35	121.1	C84—C83—H83	119.5
C36—C35—H35	121.1	C82—C83—H83	119.5
C35—C36—C37	121.9 (12)	C83—C84—C85	120.6 (10)
C35—C36—H36	119.0	C83—C84—H84	119.7
C37—C36—H36	119.0	C85—C84—H84	119.7
C32—C37—C36	120.5 (11)	C84—C85—C86	119.4 (10)
C32—C37—H37	119.7	C84—C85—H85	120.3
C36—C37—H37	119.7	C86—C85—H85	120.3
C43—C38—C39	117.4 (9)	C81—C86—C85	121.4 (9)
C43—C38—Sn2	124.3 (8)	C81—C86—H86	119.3
C39—C38—Sn2	118.3 (8)	C85—C86—H86	119.3

### Hydrogen-bond geometry (Å, °)

**Table:**

*D*—H···*A*	*D*—H	H···*A*	*D*···*A*	*D*—H···*A*
C6—H6···O6	0.98	2.48	3.417 (10)	160
C49—H49···O4	0.98	2.54	3.475 (10)	160

## References

[bb1] Allen, F. H., Kennard, O., Watson, D. G., Brammer, L., Orpen, A. G. & Taylor, R. (1987). *J. Chem. Soc. Perkin Trans. 2*, pp. S1–19.

[bb2] Gielen, M., Vanbellinghen, C., Gelan, J. & Willem, R. (1988). *Bul. Soc. Chim. Belg.* **97**, 873–878.

[bb3] Sheldrick, G. M. (1996). *SADABS* University of Göttingen, Germany.

[bb4] Sheldrick, G. M. (2008). *Acta Cryst.* A**64**, 112–122. 10.1107/S0108767307043930 18156677

[bb5] Siemens (1996). *SMART* and *SAINT* Siemens Analytical X-ray Instruments Inc., Madison, Wisconsin, USA.

[bb6] Win, Y. F., Teoh, S. G., Teh, J. B.-J., Fun, H.-K. & Zakaria, L. (2007). *Acta Cryst.* E**63**, m323–m325.

